# Early detection of mental health disorders using machine learning models using behavioral and voice data analysis

**DOI:** 10.1038/s41598-025-00386-8

**Published:** 2025-05-13

**Authors:** Sunil Kumar Sharma, Ahmed Ibrahim Alutaibi, Ahmad Raza Khan, Ghanshyam G. Tejani, Fuzail Ahmad, Seyed Jalaleddin Mousavirad

**Affiliations:** 1 Department of Information Systems, College of Computer and Information Sciences, Majmaah University, 11952 Majmaah, Saudi Arabia; 2 King Salman Center for Disability Research, 11614 Riyadh, Saudi Arabia; 3 Department of Computer Engineering, College of Computer and Information Sciences, Majmaah University, 11952 Majmaah, Saudi Arabia; 4 Information Technology Department, College of Computer and Information Sciences Majmaah University, Majmaah, 11952 Saudi Arabia; 5 Department of Research Analytics, Saveetha Dental College and Hospitals, Saveetha Institute of Medical and Technical Sciences, Saveetha University, Chennai, 600077 India; 6 Department of Industrial Engineering and Management, Yuan Ze University, Taoyuan, 320315 Taiwan; 7 Applied Science Research Center, Applied Science Private University, Amman, 11937 Jordan; 8 Respiratory Care Department, College of Applied Sciences, Almaarefa University, Diriya, Riyadh, Saudi Arabia; 9 Department of Computer and Electrical Engineering, Mid Sweden University, Sundsvall, Sweden

**Keywords:** Mental health disorders, Deep learning, Behavioral data, Voice data, Machine learning, Computational science, Disability

## Abstract

People of all demographics are impacted by mental illness, which has become a widespread and international health problem. Effective treatment and support for mental illnesses depend on early discovery and precise diagnosis. Notably, delayed diagnosis may lead to suicidal thoughts, destructive behaviour, and death. Manual diagnosis is time-consuming and laborious. With the advent of AI, this research aims to develop a novel mental health disorder detection network with the objective of maximum accuracy and early discovery. For this reason, this study presents a novel framework for the early detection of mental illness disorders using a multi-modal approach combining speech and behavioral data. This framework preprocesses and analyzes two distinct datasets to handle missing values, normalize data, and eliminate outliers. The proposed NeuroVibeNet combines Improved Random Forest (IRF) and Light Gradient-Boosting Machine (LightGBM) for behavioral data and Hybrid Support Vector Machine (SVM) and K-Nearest Neighbors (KNN) for voice data. Finally, a weighted voting mechanism is applied to consolidate predictions. The proposed model achieves robust performance and a competitive accuracy of 99.06% in distinguishing normal and pathological conditions. This framework validates the feasibility of multi-modal data integration for reliable and early mental illness detection.

## Introduction

Depression is one of the mental illnesses that interfere with a person’s everyday emotions, thoughts, and behavior as well as their overall health^1^. Suicidal thoughts, disinterest, insomnia, as well as depressed mood are just few symptoms of depression which comprises 280 million people worldwide. However, the disease was misdiagnosed due to the stigma surrounding mental illness and the lack of reliable diagnostic techniques^2^. Early diagnosis is crucial for successful outcomes, even though the majority of treatment is pharmacological or therapeutic^3^. Machine Learning (ML) has been applied to detect depression and then, hybrid models integrate various ML techniques to increase accuracy. Further AI-enabled techniques shown promising results in identifying depressive symptoms include facial expression detection and EEG analysis^4^. Advances in Natural Language Processing (NLP) allow sentiment analysis to play a crucial role in the early identification of mental health issues through social media interactions, patient interviews, or therapy sessions^5^.

Applying ML techniques to large and complex datasets enables the detection of intricate patterns that traditional methods cannot identify. Utilizing wearable devices and mobile applications for continuous monitoring facilitates timely interventions by tracking symptoms^6^. Additionally, utilizing transfer learning enhances model adaptability to diverse populations, thereby improving universality and accessibility^7^. Later, studies for depression using ML have been conducted for analyzing data concerning physiological signals, text-based interactions, voice patterns, facial expressions, and social media activity to estimate a mental health condition and detect depressive symptoms^8^. Encouraging findings have been achieved with models such as Deep Learning (DL), NLP, and conventional classifiers regarding the recognition of depressive symptoms and the extraction of relevant information^9^. However, such methods have a number of constraints including algorithmic biases that lead to incorrect predictions for many populations. Besides, problems in terms of ethical challenges regarding consent as well as usage of data, and privacy challenges imposed on by the sensitive nature of mental health data^10^. It is also challenging for models to generalize well due to the complex, multi-faceted nature of depression: biological, psychological, and social factors all influence this affective disorder^11^.

AI-empowered speech as well as behaviour pattern detection in behavioural and voice data analysis provides a scalable, non-invasive method to monitor depression^12^. However, these methods still face challenges, including algorithmic bias, privacy concerns, and the complexity of mental health^13^. Indeed, the need for integration with traditional treatment practices is emphasized by the fact that these technologies often lack clinical validation and have ethical, legal, as well as miscommunication problems^14^. Advances in multimodal data extraction including speech, text, and physiological signals is utilized in the context of depression diagnosis through ML to increase diagnosis accuracy as well as generate more individualized predictions of future treatment designs for upcoming studies^15^. Still, sustainable, ethical, and therapeutically integrated solutions are needed to address the lack of standardized datasets, the risk of false positives or negatives, and the paucity of clinical validation, all of which hinder their practical application^16^. For this reason, this paper introduces a NeuroVibeNet for better detection and classification. The key contributions are:

- To assess diverse features, 2 distinct datasets for behavioral and voice data are used and both are pre-processed individually to retain the individuality of each data.
- To effectively handle the behavioral data, an IDTW is proposed for temporal pattern analysis.
- After combining the features from 2 datasets, an MRFE is applied to reduce the feature dimensionality.
- To significantly detect the early symptoms, an efficient NeuroVibeNet is proposed to identify either the data is normal or abnormal.

This article is structured as a recent literature on mental illness detection in Section II. Implemented framework and the description are given in Section III. Results and the significance are presented in Section IV. Section V ends the research.

## Literature study

### Recent research

In 2024, Zhang^17^ suggested CNN and LSTM models to identify adolescents with depression having early symptoms. The electronic health records of over 50,000 teenagers were trained on a sizable clinical dataset utilizing neuroimaging data. 92% F1, and 97% AUC, was recorded as their impressive performance.

In 2024, Satapathy et al.^18^ evaluated the performance of various algorithms in the classification of sleep disorders like insomnia, sleep apnea, and narcolepsy. The models captured deep patterns and dependencies inside EEG data to permit earlier detection and more accurate determination. Notably, CNN and RNN outperformed the traditional algorithms for sleep disorders.

In 2024, Hossain et al.^19^ suggested an automatic facial expression detection system using quantum and traditional DL models with video, sequential, and static facial images from medical data to track emotions. The five-step method improved performance by combining scores from quantum and traditional DL models.

In 2024, Diwakar and Raj^20^ proposed a text classification model employing DistilBERT to classify mental health disorders in an automated manner. Three disorders such as autism, BPD, as well as anxiety were tested. In addition, the potential link between the microbiota and mental health and the gut-brain axis were explored. A balanced dataset with 500 samples per class provided an accuracy of 96% for this model.

In 2024, Peristeri et al.^21^ proposed a AI-based model that differentiated children with Autism Spectrum Disorder (ASD) using gradient boosting (XGBoost). By employing NLP techniques, features were extracted from storytelling data for 52 children with usual development and 68 children with ASD. Their behavioural targets developed a significant difference between the two groups by the produced ML models.

In 2024, Upadhyay et al.^22^ employed a stacking SVM ensemble approach for the analysis of behavioral data to have a better chance in early detection of Persistent Depression Disorder (PDD). From Experimentation, PDD was found mostly present among middle-class students studying nontechnical subjects and among the rural students belonging to higher and lower income groups.

In 2024, Revathy et al.^23^ demonstrated Dynamically Stabilized Recurrent Neural Network (DSRNN) for more accuracy on extracting features and providing diagnostic capabilities for mental disease problems. The OSMI dataset was employed to extract the critical features. The frequency component relations between patients and healthy persons were concentrated. Table 1 presents the recent literature on mental illness detection using various methods.

**Table 1:** Recent literature on mental illness detection using various methods.

Authors/Year	Methods	Aim	Advantages	Limitations
Zhang in 2024^17^	Hybrid CNN-LSTM	To detect early warning signs of depression in teenagers	High performance with 95% accuracy,	Required large clinical datasets for effective training
Satapathy et al. in 2024^18^	CNN, and RNN	To identify sleep disorders like insomnia, sleep apnea, and narcolepsy	DL models outperform traditional algorithms	Complex EEG data required significant preprocessing
Hossain et al. in 2024^19^	Classical and quantum DL models	To analyse facial expressions to detect emotions in healthcare data	Improved performance on benchmark datasets Fusion of multiple models	Limited by quality of facial image data
Diwakar & Raj in 2024^20^	DistilBERT-based text classification	To automate diagnosis of anxiety, BPD, and autism	Achieved 96% accuracy with balanced dataset	Failed to fully capture complex, diverse symptoms of mental health
Peristeri et al. in 2024^21^	NLP, ML	To distinguish ASD from typically-developing children	Achieved 96% accuracy	Need to generalize well across all age groups or demographics
Upadhyay et al. in 2024^22^	Stacking SVM ensemble approach	To improve early diagnosis of PDD	Achieved 89.4% accuracy	Need to account for all socio-economic factors influencing PDD
Revathy et al. in 2024^23^	DSRNN, feature extraction	To diagnose mental illness disorders	High accuracy (98% and 99.5%)	Complexity in handling diverse age groups and data variability

### Problem Statement

Mental illness is a growing global health concern, yet its detection remains challenging due to subjective diagnostic methods, and the lack of reliable tools. Traditional diagnostic approaches often fail to capture subtle behavioural and vocal patterns that signify early mental health deterioration. ML offers a promising solution by analyzing large, multi-modal datasets such as behavioural and voice data to identify complex, non-linear patterns that are imperceptible to human observation. The integration of ML-based models can transform mental health diagnostics, enabling earlier intervention and reducing the risk of severe complications. ML-driven mental illness detection offers several advantages including automated analysis of complex data, the ability to process multi-modal inputs, and scalability for continuous monitoring through wearable devices and mobile applications. These systems can enhance the objectivity of diagnosis and improve accessibility to mental health services. However, challenges persist, such as algorithmic bias, the need for high-quality and diverse datasets, and ensuring robust privacy and security for sensitive mental health data. Additionally, a lack of clinical validation and the complexity of mental health conditions pose barriers to the practical implementation of these systems. Addressing these challenges is essential to realize the full potential of ML in mental health diagnostics.

## A novel mental illness disorders detection model

### Proposed architecture

**Fig. 1:**
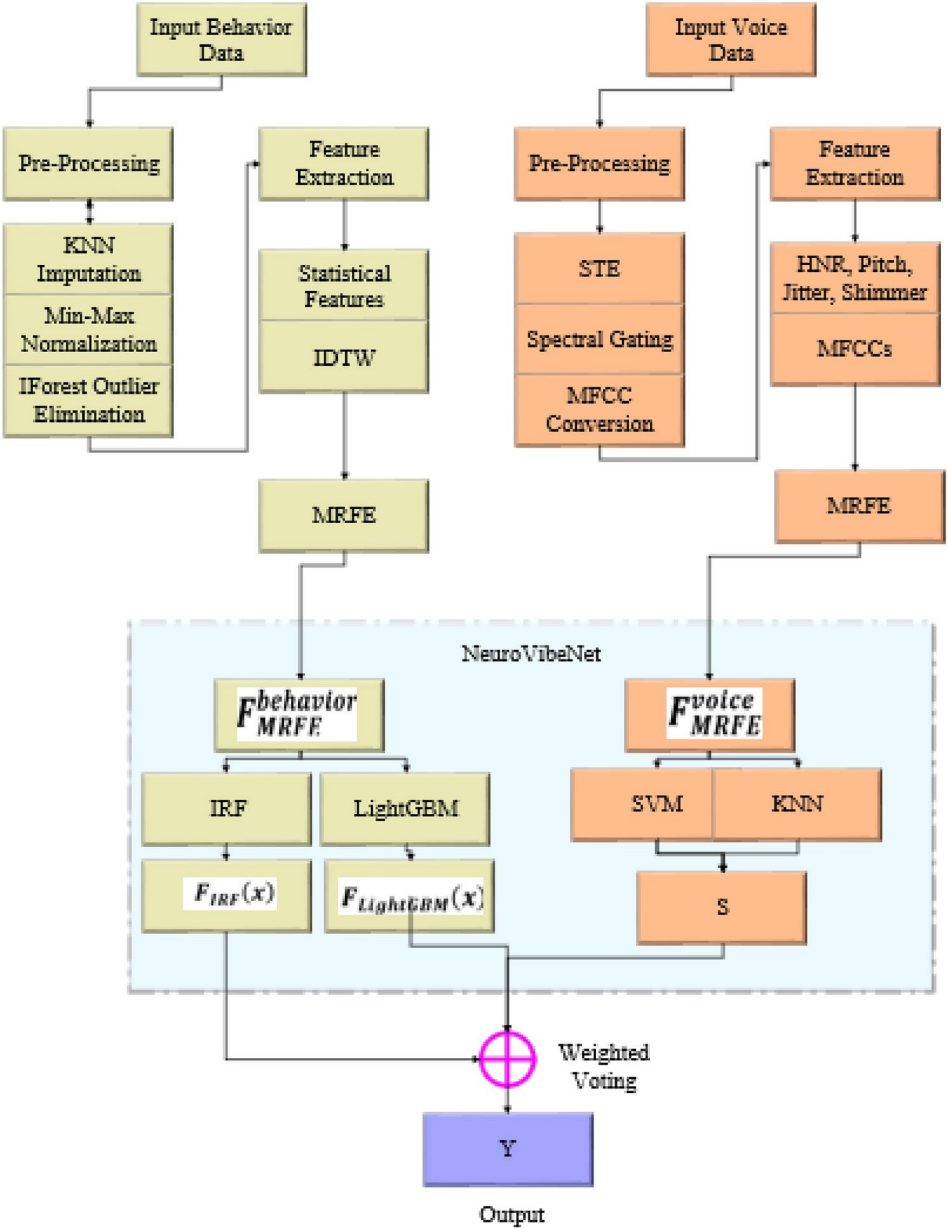
Block diagram of proposed model.

Figure 1 outlines the overview of developed early detection of mental illness disorders framework. This study uses speech and behavioural data to train a network that can handle multi-modal data. The detection step uses two different datasets that have been pre-processed separately. KNN imputation for missing data, Min–Max normalization, and IForest outlier elimination are used for behavioural data. Voice data is segmented using STE, and noise is reduced via spectral gating. Behavioural data also uses IDTW for temporal pattern analysis and statistical techniques mean, variance, and skewness for time-series feature extraction. HNR extraction, pitch, jitter, shimmer, and MFCCs are also used for extracting voice data. Furthermore, feature selection using a proposed MRFE technique is applied. Finally, the suggested NeuroVibeNet evaluates the data to determine if it is normal or pathological. The proposed NeuroVibeNet is built with IRF, and LightGBM for behavioral data and hybrid SVM and KNN for voice data. Finally, a weighted voting is applied to accomplish the output.

### Datasets description

For early detection of mental illness detection, 2 distinct datasets are used in this research such as Mental Disorder Classification for behavioral data in https://www.kaggle.com/datasets/cid007/mental-disorder-classification and Multi-modal Open Dataset for Mental-disorder Analysis (MODMA) Dataset for voice data in https://modma.lzu.edu.cn/data/index/. In order to align data in both datasets into an assessible format, both the datasets are arranged and concatenated as follows.

1. *Sort Each Dataset by Labels:* Ensure both the behavioral and voice datasets are sorted by their respective labels (e.g., all rows with label 0 followed by all rows with label 1). This maintains consistency when combining.
2. *Index Matching for Concatenation:* Align the datasets row-wise to ensure that ${i}^{th}$ row of the behavioral dataset corresponds to ${i}^{th}$ row of the voice dataset for the same label.
3. *Concatenate Datasets:* Once sorted and aligned, concatenate the datasets along the column axis by creating a unified dataset where features from both sources are combined with the label column preserved.

This ensures the final dataset is coherent with correctly paired data from behavioral and voice.

Sensitive behavioral and voice data utilization within NeuroVibeNet creates fundamental ethical challenges related to protection of user privacy together with data confidentiality and obtaining proper consent. Secure data storage and transmission for participants involve implementing strict encryption protocols together with data anonymization procedures that protect identity information. The process of data collection functions under ethical standards that require both consent from participants and IRB approval. User rights and personal information protection against unauthorized misuse or access become possible through GDPR and HIPAA compliance practices^24,25^. The framework’s responsible AI deployment commitment gets strengthened by periodic ethical reviews and audit processes.

## Description for MODMA

The research employs two publicly available datasets namely Mental Disorder Classification and Multi-modal Open Dataset for Mental-disorder Analysis (MODMA) which are commonly used by researchers. The Mental Disorder Classification dataset includes behavioral data from participants of different demographics who exhibit depression, anxiety and bipolar disorder symptoms with equal distribution among age groups and genders. The dataset offers voice data through multiple modalities which includes recorded speech from participants with various mental health conditions (depression, bipolar disorder, schizophrenia) and normal control subjects who provided samples in standardized conditions for quality assurance. We utilized data normalization and outlier removal techniques for bias prevention then employed stratified sampling during training to achieve balanced class representations. The model’s ability to generalize across different data subsets was evaluated through cross-validation which minimized both overfitting and bias-related risks.

There are two advantages NeuroVibeNet brings over single-modal conventional evaluations through its combination of attitudinal and vocal data elements which enhance both diagnostic performance and modeling stability. The identification of mental health issues depends on multiple symptoms which affect both verbal and physical expressions of human behavior. A single data source has limited capabilities to record the complex clinical scenario leading to suboptimal or unreliable diagnostic outcomes^26^. Behavioral data reveals mental health disorder suspicions through the observation of sleep patterns together with measurement of exercise activity and social interaction and lifestyle behaviors. Voice data contains tone, pitch and speech tempo and vocal prosody which science demonstrates links to emotional states such as stress and anxiety and depression. Each single data source provides essential yet limited information when used independently. The system NeuroVibeNet exploits the different capabilities of these two modalities through their joint operation. The detection of immediate emotional states through voice data outperforms behavioral data which reveals long-term behavioral patterns in patient activities. The merging of sources enables a deep analysis to provide an extensive understanding of mental states. The evaluation of early diagnosis along with timely interventions heavily depends on cross-modality signals. Voice characteristics reveal emotional distress even though a person appears to perform regular activities according to behavioral data analysis. The additional data source functions as an error-reduction mechanism to prevent incorrect classifications when one of the inputs contains noise or missing information or lacks clarity^24,25^. NeuroVibeNet achieves its beneficial outcome through models that apply Improved Random Forest (IRF) and LightGBM for structured behavioral data and hybrid SVM and KNN combinations for acoustic speech analysis. The weighted voting process combines predictions synergistically to minimize the individual biases and eliminate variances found in separate models. NeuroVibeNet demonstrates its ability to work with diverse clinical and demographic groups through its final fusion design which leads to its 99.06% accuracy rate. The multi-modal approach of NeuroVibeNet provides enhanced management of real-world complications because mental health presentations naturally vary which enables early and accurate mental disease diagnosis.

###  Preprocessing

It is carried out to ensure both behavioral and voice data are clean, consistent, and ready for analysis by removing noise, handling missing values, and normalizing feature scales.

*1) For Behavioral Data:*Since behavioral data is text-based, KNN imputation^27^is used for handling missing data, Min–Max normalization^28^is applied to scale the features within a defined range, and IForest^29^ is used for outlier elimination. This ensures that the data is clean, consistent, and suitable for model training.

*a) KNN Imputation:* It is used to handle missing data by filling in missing values based on the values of their nearest neighbors. The algorithm works by finding the $^{\prime}K^{\prime}$ closest data points to the instance with missing values and then using their feature values to estimate the missing one. Given a dataset $X$ with some missing values, the goal is to predict the missing value ${x}_{missing}$. Calculate the distance $D\left({x}_{i},{x}_{j}\right)$ between data points ${x}_{i}$ and ${x}_{j}$ using Euclidean distance as stated in Eq. (1), in which ${x}_{ik}$ and ${x}_{jk}$ defines the corresponding feature values of data points ${x}_{i}$ and ${x}_{j}$.

1 $$D\left({x}_{i},{x}_{j}\right)=\sqrt{\sum_{k=1}^{n}{\left({x}_{ik}-{x}_{jk}\right)}^{2}}$$

Now, the nearest neighbors are selected based on $K$ nearest neighbors (data points with the smallest distances). For the missing value in feature $k$, the imputed value ${x}_{missing}$ is calculated by averaging the corresponding values of the nearest neighbors as shown in Eq. (2), in which ${x}_{neignbor,k}$ signifies value of feature $k$ for each of $K$ nearest neighbors.

2 $${x}_{missing}=\frac{1}{K}\sum_{i=1}^{K}{x}_{neignbor,k}$$

* b) *Min–Max normalization: It is a feature scaling technique that transforms data into a specific range between $\left(\text{0,1}\right)$. Besides, it is used to ensure that all features contribute equally to the model by scaling them to a uniform range to avoid bias towards variables with larger numerical ranges. Given a feature $x$ in the dataset with a minimum value ${x}_{min}$ and a maximum value ${x}_{max}$, the normalized value ${x}^{\prime}$ is calculated in Eq. (3).

3 $$x^{\prime} = \frac{{x - x_{min} }}{{x_{max} - x_{min} }}$$

*c) Iforest*: It is an anomaly detection algorithm that isolates outliers instead of profiling normal data. It works by creating multiple decision trees where data points that are easy to isolate (i.e., those far from the rest of the data) are considered outliers. The number of splits (or partitions) required to isolate a data point is called its path length. Given a data point $x$, the path length $l\left(x\right)$ specifies the number of edges traversed in the isolation tree to isolate the point. Moroever, for a data point $x$, the path length $l\left(x\right)$ is computed as the number of splits required to isolate $x$ in the decision tree. The average path length for a point is calculated over multiple trees. The anomaly score $s\left(x\right)$ of a data point $x$ is calculated based on the average path length $l\left(x\right)$ across all trees as defined in Eq. (4), where $c\left(n\right)$ refers to average path length of a point in a binary search tree and is defined in Eq. (5).

4 $$s\left(x\right)={2}^{-\frac{l\left(x\right)}{c\left(n\right)}}$$

5 $$c\left(n\right)=2\left(\text{ln}\left(n-1\right)+r\right)$$

Here, $n$ addresses data point count in the dataset, and $r$ states Euler’s constant (0.5772). Finally, the anomaly score interpretation is calculated based on points with a higher anomaly score (closer to 1) are considered anomalies, and points with lower scores (closer to 0) are considered normal.

*2) For Voice Data:*For voice data, segmentation is performed using STE^30^to identify speech activity, followed by noise reduction through spectral gating^31^to eliminate background noise. The cleaned audio is then converted into MFCCs^32^ to capture key features for further analysis.

*a) STE:* It is used to segment an audio signal into frames. The energy of a signal is calculated over a short period of time to capture changes in the signal’s amplitude. It is typically used to detect speech activity and silence, helping to divide the audio into meaningful segments. For an audio signal $x\left(t\right)$, the short-time energy is computed over a window of size $N$ and a shift of $S$ between successive windows as given in Eq. (6), where $E\left(t\right)$ states energy at time frame $t$, $x\left(t+n\right)$ signifies signal at time $\left(T+n\right)$, and $n$ represents the sample points within the window.

6 $$E\left(t\right)=\sum_{n=0}^{N-1}{\left|x\left(t+n\right)\right|}^{2}$$

*b) Noise Reduction using Spectral Gating:* It is a noise reduction technique that works by identifying and removing noise components from the audio signal. The idea is to apply a gate to the frequency spectrum to attenuate components that correspond to noise (usually those with low amplitude or energy) while preserving speech-related components. The audio signal $X\left(f,t\right)$ in the frequency domain is represented by its Short-Time Fourier Transform (STFT). Spectral gating involves computing the STFT, and applying spectral gating. Equation (7) shows the STFT computation of the signal, in which $X\left(f,t\right)$ indicates STFT of the signal at frequency $f$ and time $t$, $x\left(t+n\right)$ states audio signal at time $\left(t+n\right)$, and $w\left(n\right)$ represents windowing function applied to the signal.

7 $$X\left(f,t\right)=\sum_{n=0}^{N-1}x\left(t+n\right)\cdot w\left(n\right)\cdot {e}^{-j2\pi ft}$$

A gate (threshold $Th$) is applied to each frequency bin. If the magnitude of a frequency component $\left|X\left(f,t\right)\right|$ s below a certain threshold (which corresponds to noise), then it is attenuated or set to zero as shown in Eq. (8), where ${X}_{gated}\left(f,t\right)$ defines result after noise reduction, and the threshold $Th$ is determined based on the noise characteristics and typically varies with frequency.

8 $${X}_{gated}\left(f,t\right)=\left\{\begin{array}{cc}X\left(f,t\right)& if \left|X\left(f,t\right)\right|\ge Th\\ 0& o.w\end{array}\right.$$

*c) Conversion to MFCCs:* It is widely used features for speech recognition and processing. They represent the power spectrum of the audio signal and capture the important characteristics of the speech signal by approximating how humans perceive sound frequencies. First, apply the STFT to the gated signal ${X}_{gated}\left(f,t\right)$ to obtain its power spectrum as expressed in Eq. (9).

9 $$P\left(f,t\right)={\left|{X}_{gated}\left(f,t\right)\right|}^{2}$$

A set of triangular filters is applied to the power spectrum to map frequencies to the Mel scale. The Mel scale approximates the human ear’s response to different frequencies, which is non-linear in higher frequencies. The Mel filter bank is applied in Eq. (10), where ${M}_{m}\left(t\right)$ addresses Mel-scaled power spectrum at frequency band $m$ and time $t$, and ${h}_{m}\left(f\right)$ states response of ${m}^{th}$ Mel filter at frequency f.

10 $${M}_{m}\left(t\right)=\sum_{f}{h}_{m}\left(f\right)\cdot P\left(f,t\right)$$

Finally, the logarithm of the Mel-scaled spectrum is taken, and a Discrete Cosine Transform (DCT) is applied to extract the MFCCs as illustrated in Eq. (11), in which $MFC{C}_{n}\left(t\right)$ explains ${n}^{th}$ MFCC at time $t$, ${M}_{m}\left(t\right)$ states Mel-scaled power spectrum from the previous step, and $n$ indexes the number of MFCCs (12–13 coefficients).

11 $$MFC{C}_{n}\left(t\right)=\sum_{m=1}^{M}log\left({M}_{m}\left(t\right)\right)\cdot \text{cos}\left[\frac{\pi n}{M}\left(m-\frac{1}{2}\right)\right]$$

The preprocessing methods used for behavioral and voice data maintain consistent and high-quality data by handling standard data quality problems including missing values and different feature scales and outliers and noise which lead to better model performance. KNN imputation substitutes behavioral data values through data point similarity analysis so that the underlying data distribution remains intact. Min–Max normalization applies feature scale standardization to create equal variable influence on model learning while controlling factors that have wide numerical ranges. The IForest method detects and eliminates data outliers that would distort analysis results or training models thus improving data reliability. STEnet divides audio data into usability sections by detecting speech segments before spectral gating cleans noises to enhance audible signal clarity. The cleaned signal gets transformed into MFCCs which extracts crucial speech characteristics from the data. These methodologies transform raw data into orderly and noise-free sets that machine learning can effectively use which produces accurate models with better generability and robustness.

### Feature extraction

This procedure involves deriving meaningful patterns and attributes from raw data to enhance model training and prediction. Similarly, voice data incorporates MFCCs, pitch, jitter, shimmer, and HNR for capturing speech characteristics.

*1) Behavioral d*ata: Behavioral data uses statistical measures like mean, variance, skewness^33^ and proposed IDTW for temporal analysis.

*a)* Statistical Analysis: Time-series feature extraction involves computing statistical metrics from the data to capture its essential characteristics. The mean represents the average value of the time-series data over a given period as described in Eq. (12), where ${x}_{i}$ points to value at ${i}^{th}$ time step, and $\aleph$ refers to total time step count.

12 $$\mu =\frac{1}{\aleph }\sum_{i=1}^{\aleph }{x}_{i}$$

In Eq. (13), variance measures the spread of the time-series data around the mean, indicating variability.

13 $${\sigma }^{2}=\frac{1}{\aleph }\sum_{i=1}^{\aleph }{\left({x}_{i}-\mu \right)}^{2}$$

In Eq. (14), skewness measures the asymmetry of the time-series data distribution, where $S>0$ and $S<0$ indicate right-and left-skewed distribution, respectively.

14 $$S=\frac{\left(\frac{1}{\aleph }\sum_{i=1}^{\aleph }{\left({x}_{i}-\mu \right)}^{3}\right)}{{\left(\frac{1}{\aleph }\sum_{i=1}^{\aleph }{\left({x}_{i}-\mu \right)}^{2}\right)}^\frac{3}{2}}$$

*b) Temporal Pattern Analysis Using Proposed IDTW:*Normally, Dynamic Time Warping (DTW)^34^ is a method used to measure similarity between two temporal sequences that may vary in time or speed. The proposed IDTW optimizes this process by integrating constraints and penalties to handle noise and align sequences more robustly. The distance between two sequences $A=\left\{{a}_{1},{a}_{2},\dots ,{a}_{N}\right\}$ and $B = \left\{{b}_{1}, {b}_{2}, \dots , {b}_{M}\right\}$ is defined in Eq. (15), in which $D\left(i,j\right)$ indicates cumulative distance at point $\left(i,j\right),$ and $\left|{a}_{i}-{b}_{j}\right|$ signifies local cost between ${a}_{i}$ and ${b}_{j}$.

15 $$D\left(i,j\right)=\left|{a}_{i}-{b}_{j}\right|+\text{min}\left\{D\left(i-1,j\right),D\left(i,j-1\right),D\left(i-1,j-1\right)\right\}$$

At this point, weighting factors are applied by incorporating weights to emphasize specific time points as specified in Eq. (16), in which $w\left(i,j\right)$ denotes weighting matrix.

16 $$D\left(i,j\right)=w\left(i,j\right)\cdot \left|{a}_{i}-{b}_{j}\right|+\text{min}\left\{D\left(i-1,j\right),D\left(i,j-1\right),D\left(i-1,j-1\right)\right\}$$

Equation (17) add penalties for skipping too many consecutive points, where $\alpha$ denotes penalty factor, and $p$ indicates skip penalty.

17 $$D\left(i,j\right)=\left|{a}_{i}-{b}_{j}\right|+\alpha \cdot \text{min}\left\{D\left(i-1,j\right)+p,D\left(i,j-1\right),p,D\left(i-1,j-1\right)\right\}$$

The optimal path $P=\left\{\left({i}_{1},{j}_{1}\right),\left({i}_{2},{j}_{2}\right),\dots \right\}$ is found by backtracking the minimal cumulative cost from $D\left(N,M\right)$ to $D\left(\text{1,1}\right)$ providing the best alignment of sequences $A$ and $B$. Finally, a feature vector ${V}_{behavior}$ is developed in this stage. Algorithm 1 explain the developed IDTW.

**Algorithm. 1:**
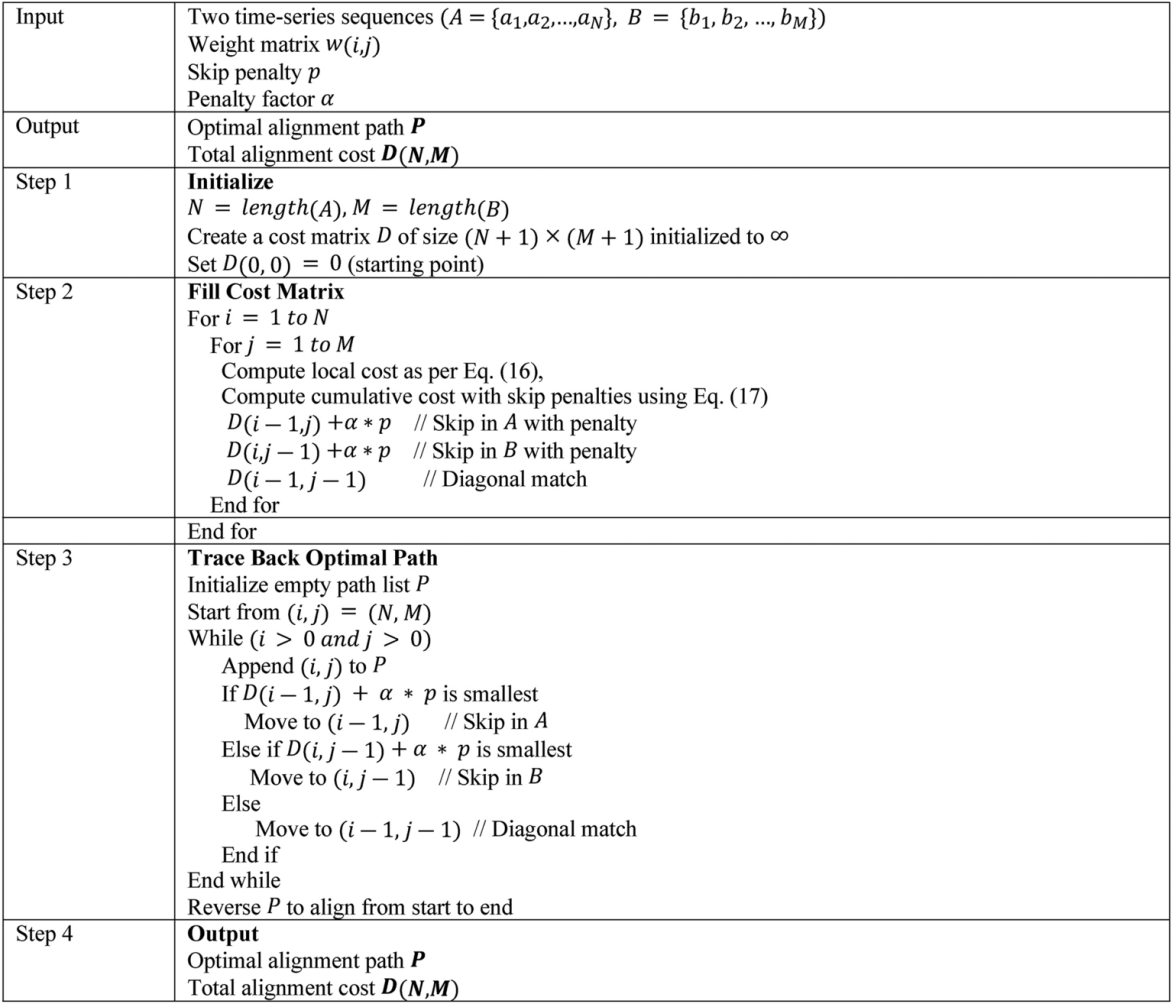
Pseudocode of Developed IDTW

*2) Voice data feature extraction:*It includes pitch, jitter, shimmer, HNR, and MFCCs^35^. These feature are extracted to capture speech variability and irregularities.

*a) Pitch:* It represents the perceived frequency of a voice, indicating how high or low the voice sounds. It is computed as the inverse of the time between two successive glottal closures in a speech signal as defined in Eq. (18), where ${T}_{0}$ states fundamental period of the signal.

18 $$Pitch\left({f}_{0}\right)=\frac{1}{{T}_{0}}$$

*b) Jitter:* It quantifies the cycle-to-cycle variation in pitch by reflecting the instability in vocal fold vibration as given in Eq. (19), where ${T}_{i}$ and ${T}_{i+1}$ denote consecutive fundamental periods, and $N$ address the total cycle count.

19 $$Jitter=\frac{1}{N-1}\sum_{i=1}^{N-1}\left|\frac{{T}_{i}-{T}_{i+1}}{{T}_{i}}\right|$$

*c) Shimmer:* It measures the cycle-to-cycle variation in amplitude by indicating vocal intensity instability as represented in Eq. (20), in which ${A}_{i}$ and ${TA}_{i+1}$ denote amplitudes of consecutive glottal cycles.

20 $$Shimmer=\frac{1}{N-1}\sum_{i=1}^{N-1}\left|\frac{{A}_{i}-{A}_{i+1}}{{A}_{i}}\right|$$

*d) HNR:* It quantifies the ratio of harmonic components to noise components in a voice signal to indicate vocal clarity and quality as signified in Eq. (21), in which ${P}_{harmonic}$ means to power of the harmonic signal, and ${P}_{noise}$ specifies the power of the noise signal.

21 $$HNR=10\cdot {\text{log}}_{10}\left(\frac{{P}_{harmonic}}{{P}_{noise}}\right)$$

*e) MFCCs:* Let the preprocessed MFCC matrix $M$ has dimensions $F\times T$, where $F$ states coefficient count, and $T$ indicates frame count. Compute mean $\left(MFCC\_{\mu }_{f}\right)$, and variance $\left(MFCC\_{\sigma }_{f}^{2}\right)$ as shown in Eq. (22), and (23), and higher-order moments (skewness $\left({S}_{f,1}\right)$, and kurtosis $\left({S}_{f,2}\right)$) for each coefficient across frames in Eq. (24), and (25) in order.

22 $$MFCC\_{\upmu }_{f}=\frac{1}{T}\sum_{t=1}^{T}M\left[f,t\right]$$

23 $$MFCC\_{\upsigma }_{f}^{2}=\frac{1}{T}\sum_{t=1}^{T}{\left(M\left[f,t\right]-MFCC\_{\upmu }_{\text{f}}\right)}^{2}$$

24 $${S}_{f,1}=\frac{1}{T}\sum_{t=1}^{T}{\left(M\left[f,t\right]-\frac{MFCC\_{\upmu }_{f}}{MFCC\_{\upsigma }_{f}^{2}}\right)}^{3}$$

25 $${S}_{f,2}=\frac{1}{T}\sum_{t=1}^{T}{\left(M\left[f,t\right]-\frac{MFCC\_{\upmu }_{f}}{MFCC\_{\upsigma }_{f}^{2}}\right)}^{4}-3$$

These statistical features are used to reduce the dimensionality of the MFCC features and represent the temporal dynamics of the speech signal more succinctly. Finally, a feature vector ${V}_{voice}$ is created at this phase.

### *Feature Selection* via *proposed MRFE*

Generally, RFE^36^ is a feature selection method that works by recursively removing the least significant features based on a ranking (weight) associated with each feature. In the context of proposed MRFE, the process is modified to consider feature weights for more precise elimination by ensuring the features contribute less to the predictive model are gradually removed. Start with all features and assign initial weights ${\omega }_{i}$ for each feature based on some criterion (feature importance, statistical relevance). Moreover, compute a score for each feature using its weight ${\omega }_{i}$. The score is typically the rank of the weight ${\omega }_{i}$, i.e., the higher the weight, the more important the feature as shown in Eq. (26), where $Scor{e}_{i}$ represents the importance ranking of feature $i$, based on the weight ${\omega }_{i}$, and $Rank\left({\omega }_{i}\right)$ assigns a rank based on the magnitude of ${\omega }_{i}$.

26 $$Scor{e}_{i}=Rank\left({\omega }_{i}\right)$$

In each iteration, the feature $f$ with the lowest score (i.e., the least important feature) is removed. After each removal, the feature weights are recalculated to reflect the current subset of features. The process repeats until a predefined number of features is reached. The proposed MRFE iteratively remove the feature with the lowest score by updating the feature set and recalculating weights. Here, the feature vectors ${V}_{behavior}$, and ${V}_{voice}$ are processed individually, (i.e., ${V}_{behavior}$ in RF and LightGBM, and ${V}_{voice}$ by SVM and KNN). Algorithm 2 defines the implemented MRFE.

**Algorithm. 2:**
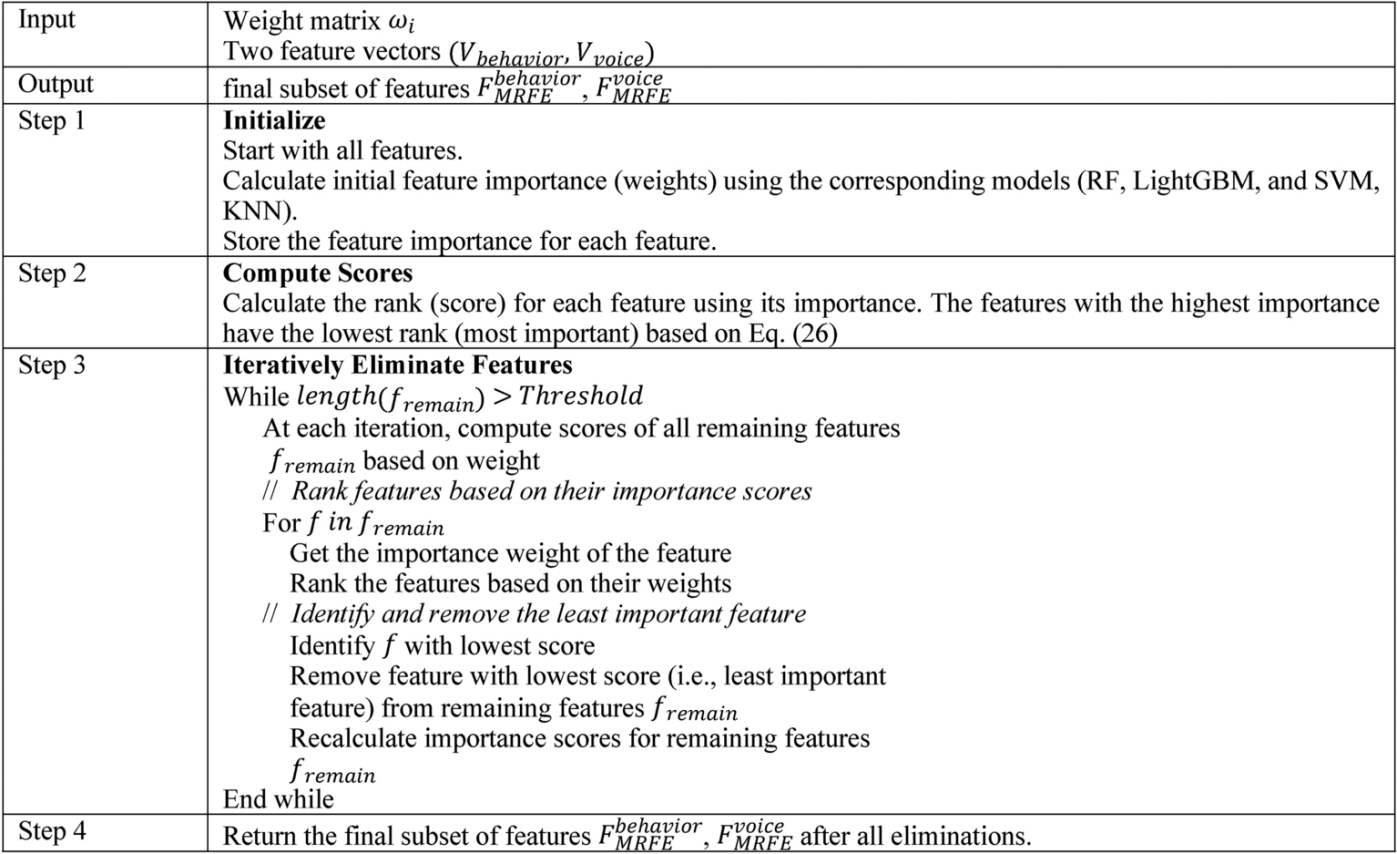
Pseudocode of Developed MRFE

### *Early detection of mental disorders *via* proposed NeuroVibeNet*

The proposed NeuroVibeNet is used to detect the mental illness disorders which is designed to focus on early symptoms. For this process, the efficacy of the traditional ML approaches is improved for RF as (IRF) and hybridization is applied to SVM and KNN models. The design of proposed NeuroVibeNet is explained in this subsection.

*1) IRF:*Generally, RF constructs multiple decision trees using bootstrapped samples and averages their predictions for classification or regression tasks^37^. The prediction is made by aggregating the individual outputs of all decision trees. In the IRF, weighted decision trees are incorporated based on performance (feature importance), and potentially uses feature sampling (more advanced tree-growing strategies) to improve accuracy and generalization. The decision trees in RF are grown independently, and each tree’s decision-making process is based on random feature subsets as defined in Eq. (27), where $F\left(x\right)$ indicates final prediction, $T$ addresses number of trees in the forest, and ${f}_{t}\left(x\right)$ points to prediction made by ${t}^{th}$ tree.

27 $${F}_{RF}\left(x\right)=\frac{1}{T}\sum_{t=1}^{T}{f}_{t}\left(x\right)$$

In IRF, each decision tree is assigned a weight based on its performance. The final prediction is then calculated by averaging the outputs of all trees with each tree’s prediction weighted by its importance as shown in Eq. (28), in which ${w}_{t}$ stands for weight assigned to ${t}^{th}$ tree based on its performance (importance).

28 $${F}_{IRF}\left(x\right)=\frac{1}{T}\sum_{t=1}^{T}{w}_{t}\cdot {f}_{t}\left(x\right)$$

The weighted sum of all tree predictions is used to generate the final output to give more importance to trees that are more accurate (informative). As for classifying mental illness, ${f}_{t}\left(x\right)$ is the class predicted by the tree.

IRF operates with weighted selection when using RF where every decision tree gets assigned a weight focusing on its performance and feature importance. IRF differs from standard RF because it gives more weight to trees which show better predictive accuracy or detect important patterns in the data. IRF improves model robustness and generalization when it applies higher weights to more informative trees particularly in noisy or imbalanced datasets that mental disorder detection involves. The weighting system decreases the impact of less important trees which results in better stability and accuracy levels. The main challenges of IRF include its higher computational requirements because of the weight-measurement procedure and its potential susceptibility to overfitting through improper weight distribution decisions based on limited training results that may degrade important RF characteristic diversity.

*2) LightGBM:*It uses Gradient Boosting to optimize decision trees iteratively by minimizing a loss function^38^. It incorporates leaf-wise tree growth for efficiency as stated in Eq. (29), where $\rho$ points to learning rate, $L$ stands for Log loss function, ${F}_{t-1}\left(x\right)$ addresses prediction from previous iteration, $h\left(x\right)$ indicates weak learner (decision tree), and $\nabla L$ signifies gradient of the loss function corresponding to predictions.

29 $${F}_{LightGBM}\left(x\right)={F}_{t-1}\left(x\right)+\rho \cdot \sum_{t=1}^{n}\nabla L\left({y}_{i},{F}_{t-1}\left({x}_{i}\right)\right)h\left(x\right)$$

*3) Hybrid SVM and KNN:*Proposed hybrid approach combines SVM’s^39^margin maximization with KNN’s^40^ local adaptability. The SVM is used for global separation and KNN refines predictions in ambiguous regions. Normally, SVM is a supervised ML algorithm used primarily for classification tasks. It aims to find the hyperplane that best separates the data into two classes while maximizing the margin between the closest data points from each class. The equation for SVM is based on optimization of minimizing the weight vector’s norm while ensuring that each data point is classified correctly as given in Eq. (30).

30 $$\underset{w}{\text{min}}\frac{1}{2}{\Vert w\Vert }^{2}$$

Subject to

$${y}_{i}^{\left(SVM\right)}\left(w\cdot {x}_{i}+b\right)\ge 1, \forall i$$

Here, $w$ points to weight vector defining the hyperplane, $b$ denotes bias, ${x}_{i}$ stands for feature vector for data point $i$, ${y}_{i}$ signifies label of data point $i$, where $yi\in \left\{-\text{1,1}\right\}$, and $\Vert w\Vert$ denotes the squared norm of the weight vector to determine the margin between the two classes. Notably, the goal is to maximize the margin between the support vectors of the two classes by minimizing $\frac{1}{2}{\Vert w\Vert }^{2}$. On the other hand, KNN is a non-parametric, lazy learning algorithm that classifies a data point based on the majority class of its $K$-nearest neighbors in the feature space. Moreover, KNN relies on the proximity of points to determine their class as represented in Eq. (31), in which $K$ refers to number of nearest neighbors considered for classification, ${y}_{k}$ stands for label of ${k}^{th}$ nearest neighbor, $1\left[{y}_{k}=c\right]$ addresses indicator function that returns 1 if ${k}^{th}$ neighbor belongs to class $c$, and 0 otherwise, and $argma{x}_{c}$ finds the class $c$ that appears most frequently among the $K$ nearest neighbors.

31 $${y}^{KNN}=arg\underset{c}{\text{max}}\sum_{k=1}^{K}1\left[{y}_{k}=c\right]$$

The key feature of this hybrid approach is the switching mechanism which decides whether to apply SVM or KNN based on the confidence of the model about a particular data point. The confidence is measured using the margin produced by the SVM classifier. If the margin is large enough (i.e., the data point is far from the decision boundary), SVM is used because it is more reliable in this case. However, if the margin is small (ambiguous) i.e., the data point is near the decision boundary, KNN is used because it provides better local adaptability. Furthermore, the switching criterion is based on a threshold $\delta$, which defines how close a data point is to the hyperplane. If the margin is greater than or equal to $\delta$, the model relies on the global separation capability of SVM. Otherwise, it switches to KNN for better local decision-making. Equation (32) models the switching criterion $S$, where $\left(w\cdot {x}_{i}+b\right)$ points to SVM decision boundary.

32 $$S=\left\{\begin{array}{cc}{y}_{i}^{\left(SVM\right)}& if \left(w\cdot {x}_{i}+b\right)>\updelta \\ {y}^{KNN}& if \left(w\cdot {x}_{i}+b\right)\le\updelta \end{array}\right.$$

Now, the outputs of IRF, LightGBM, and Hybrid SVM-KNN are combined using weighted voting to make the final prediction as shown in Eq. (33), in which ${w}_{i}$ points to weight assigned to ${i}^{th}$ classifier based on validation performance, ${y}_{i}$ addresses prediction from ${i}^{th}$ classifier, and $c$ stands for candidate class labels.

33 $$Y=arg\underset{c}{\text{max}}\sum_{i=1}^{n}{w}_{i}\cdot 1\left[{y}_{i}=c\right]$$

Table 2 summarizes the parameter settings of the proposed NeuroVibeNet. Algorithm 3 demonstrates the overall proposed mental illness detection framework.

**Table 2:** Parameter setting of proposed NeuroVibeNet.

Component	Parameter	Value
IRF	Number of Trees $\left(T\right)$	100
	Max Depth	20
	Min Samples Split	4
	Feature Sampling Method	Random Subsets
	Tree Weights $\left({w}_{t}\right)$	Based on feature importance
LightGBM	Learning Rate $\left(\rho \right)$	0.1
	Tree Growth	Leaf-wise
	Number of Leaves	20
	Max Depth	5
	Min Data in Leaf	10
	Boosting Type	Gradient Boosting
	Loss Function $\left(L\right)$	Log loss
Hybrid SVM-KNN	SVM Margin Threshold $\left(\delta \right)$	0.2
	SVM Kernel	RBF
	Regularization Parameter $\left(C\right)$	0.1
	Number of Neighbors $\left(K\right)$	5
	Distance Metric	Euclidean
Weighted Voting	Weights $\left({w}_{i}\right)$	Determined based on validation performance
	Decision Threshold	0.5

Hybrid SVM-KNN model has been selected for voice data because it combines both classifiers’ capabilities to enhance classification results despite difficult classification contexts. SVM creates a globally optimal hyperplane that achieves maximum class separation thus it works best when data regions are easily distinguished from each other. The decision-making capacity of SVM becomes limited when data points close to the boundary present challenges for classification. The ambiguous nature of certain cases makes KNN advantageous because it uses neighboring data proximity to improve predictions based on feature space local patterns. The algorithm selects SVM when the SVM margin indicates high confidence through its wide boundaries and activates KNN when the margin suggests low confidence to consider local patterns. The combination of SVM and KNN results in improved classification performance by minimizing errors in uncertain areas which produces a resilient system for difficult voice data analysis with its high noise levels.

The datasets required attention to class imbalance through the implementation of oversampling with synthetic data generation methods for normal-pathological data separation. Using Synthetic Minority Over-Sampling Technique (SMOTE)^29^ researchers produced synthetic pathological class samples to achieve better class distribution and reduce model bias towards normal class data. SMOTE generates new synthetic samples by drawing them from the line segments that connect minority class instances with their nearest neighbors to improve data diversity without duplicating existing points. The majority class samples were balanced through random undersampling which targeted normal class instances either redundant or situated in dense data areas. By combining SMOTE enrichment of minority cases and undersampling of majority classes the dataset obtained a balanced distribution that improved the diagnostic accuracy of the classifier. The adopted techniques decreased model bias alongside boosting its ability to recognize mental health conditions at an early stage during situations where pathological signals tend to fade from view.

NeuroVibeNet addresses overfitting and improves model generalization through L2 regularization and the combination of batch normalization and dropout techniques. The addition of a regularization term to the loss function through L2 regularization makes the model penalize weight sizes while promoting simpler models that excel in generalization tasks. Desktop normalization enhances training stability while speeding up performance through layer input normalization which reduces internal covariate shift and optimizes convergence. During training dropout actively eliminates random neurons from the network which makes it develop more stable features. Through combined application these methodologies supported NeuroVibeNet to strike the right balance between reducing model bias and controlling the variance thus enabling more consistent performances during training and validation.

The Modified Recursive Feature Elimination (MRFE) performs an enhanced feature selection procedure through iterative updates which recompute feature importance during each step. The initial step assigns weights to all behavioral and voice dataset features using importance scores calculated from RF and LightGBM and SVM and KNN models. Feature ranking takes place after weighing the features to assess the significance levels between each variable. The MRFE methodology updates its feature weights throughout the elimination process because it recognizes that feature dependencies alter after each removal step. The feature elimination process continues until a specified number of selected features reaches the predefined threshold while removing the least important feature in each iteration according to its lowest score. Repetitive recomputation of weights between iterations leads to more precise selection of features that intends to yield better model prediction and generalization abilities. The MRFE approach outperforms traditional feature selection methods through continual updates of feature importance after every elimination because it makes the model flexible to changes in relevance while reducing feature sets. The repeated weight calculation by MRFE avoids standard RFE’s dependence on static feature rankings while it detects shifting dependencies that allows for more precise context-aware feature selection. The adjustment mechanism allows the model to avoid discarding potentially important features even though they lose importance in smaller subset sizes thus resulting in robust and accurate and generalizable model.

**Algorithm. 3:**
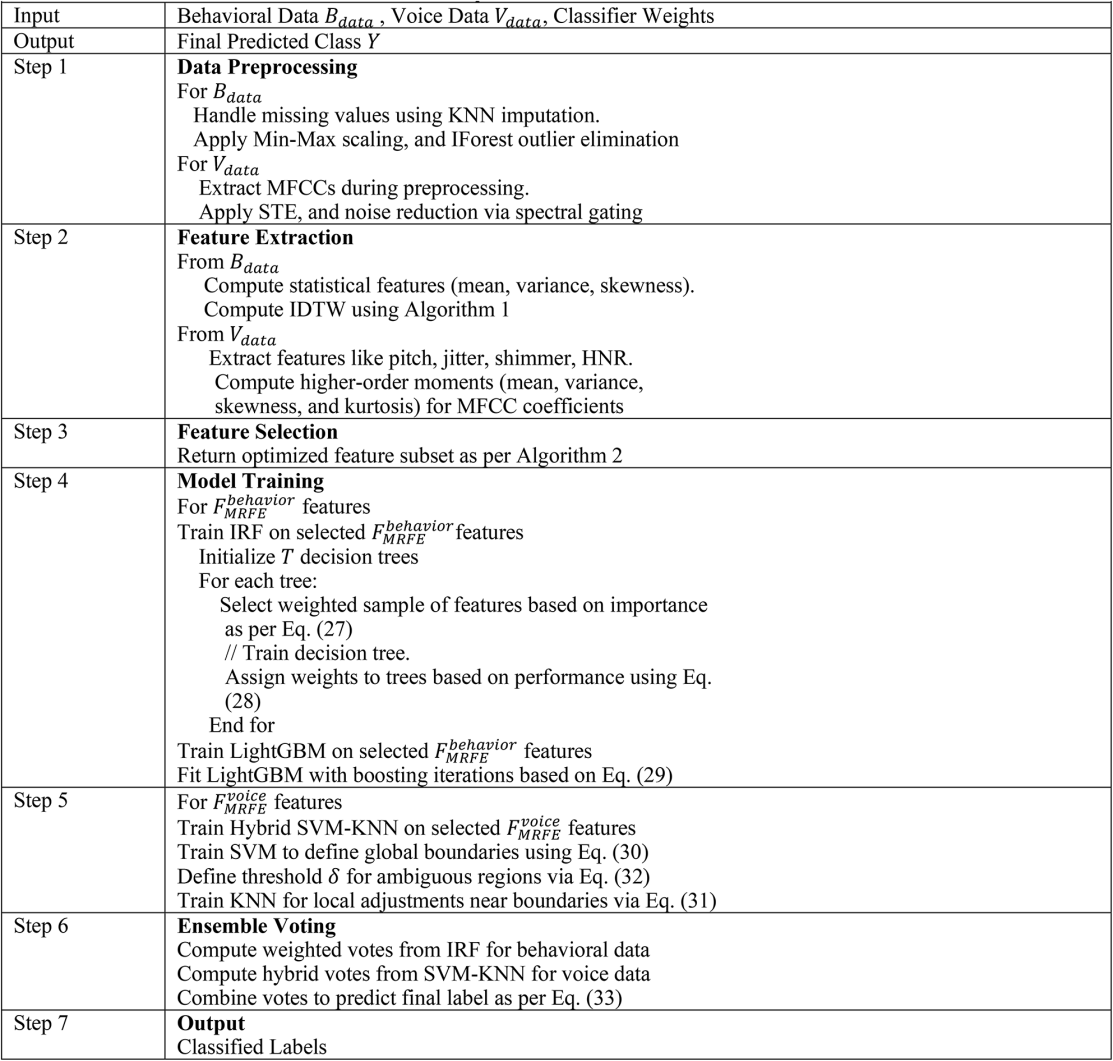
Overall Proposed Mental Illness Detection Framework

**Fig. 2:**
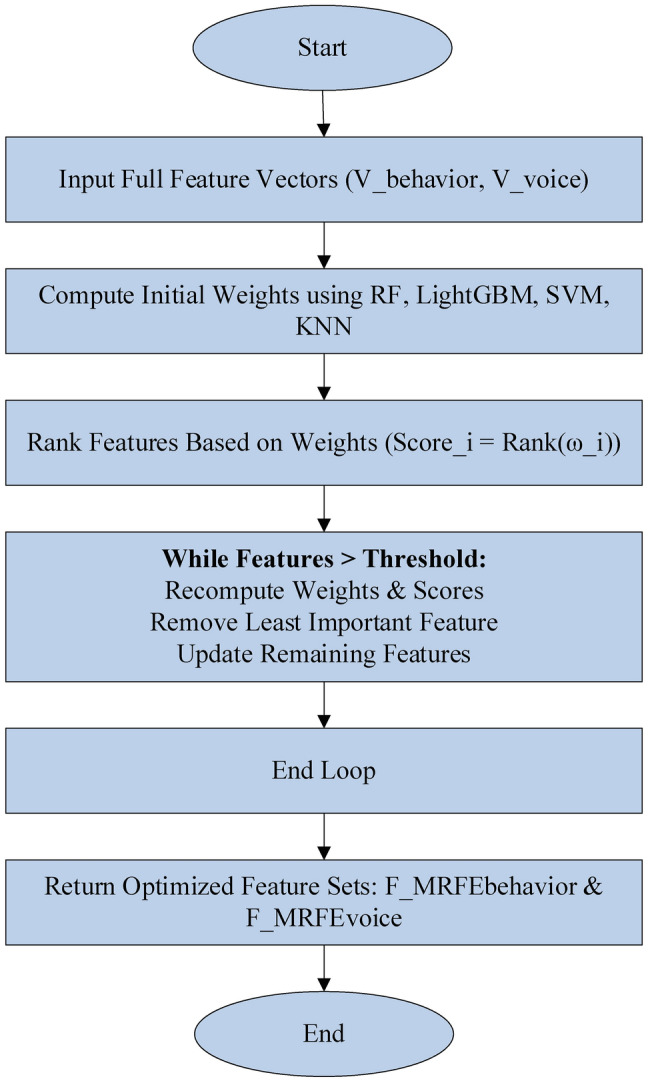
Workflow of MRFE.

The NeuroVibeNet system could reach better diagnostic power by including EEG signal processing and behavioral indicator analysis with facial emotion detection shown in Fig. 2.. The combination of various modalities allows the model to identify delicate mental disorder patterns more effectively. The system faces difficulties because it requires handling complex data together with maintaining synchronization among various data streams and needs extensive multimodal datasets for effective model training processes. Advanced fusion techniques such as late fusion and attention-based fusion should be applied to solve these challenges while domain adaptation methods would help standardize heterogeneous data sources. The combination of multimodal pretrained models together with transfer learning methods helps decrease the requirement for vast labeled dataset quantities and boosts generalization abilities. Users can apply NeuroVibeNet for other mental health detection through adding disorder-specific behavioral and voice biomarke rs to the feature extraction process. A wider implementation of this framework requires model retraining with labeled data collection from various disorders followed by processing optimization for detecting distinct patterns in behavior text and voice characteristics. By integrating NeuroVibeNet with mobile and wearable technology systems it becomes possible to conduct real-time mental health assessments in natural ecological settings. To deploy the model in real-time one must optimize its performance for fast inference along with edge computing requirements while preserving both accuracy and robustness levels.

## Simulation results

### Simulation setup

The proposed mental illness disorders detection model using the suggested NeuroVibeNet was developed via Python on Intel core® i5 processor @2.6 GHz, 16 GB RAM, 64-bit OS. For this process, 2 distinct datasets are used: Mental Disorder Classification for behavioral data, and MODMA Dataset for voice data. The efficacy of the proposed model is recorded via several performance measures such as accuracy, False Positive Rate (FPR), False Negative Rate (FNR), and Mathew’s Correlation Coefficient (MCC). For comparison, baseline models including RF, LightGBM, SVM, and KNN, and recent ML models like Logistic Regression (LR)^41^, and XGBoost^42^ are employed.

### Algorithmic analysis

Outcomes and competence of the proposed NeuroVibeNet are represented in this section. Table 3 presents a comparative analysis of the proposed NeuroVibeNet model over various ML models including RF, SVM, LightGBM, KNN, LR, and XGBoost for 80:20 learning samples with various metrics. The developed NeuroVibeNet achieves the highest accuracy (99.06%) and F1-Score (99.00%). The developed NeuroVibeNet consistently outperforms other models across all metrics by demonstrating its robustness in balancing sensitivity and specificity. Additionally, it records the lowest error rates (FPR: 0.80%, FNR: 1.10%). Among competing methods, XGBoost shows strong performance by achieving an accuracy of 97.40%. Similarly, LightGBM (96.85% accuracy) and RF (95.32%) demonstrate competitive results but lack the precision and consistency achieved by NeuroVibeNet. Besides, models like KNN (90.25%) and LR (88.90%) exhibit relatively lower performance. Thereby, it highlights the suggested NeuroVibeNet’s advantage in handling complex data and extracting meaningful insights with its innovative architecture.

**Table 3:** Performance of proposed neurovibenet using various metrics over other models for 80:20 learning samples.

Model	Accuracy	Precision	Specificity	Sensitivity	F1-Score	MCC	NPV	FPR	FNR
RF	95.32	95.00	96.10	94.80	94.90	0.89	94.50	3.90	5.20
SVM	93.74	93.20	94.00	93.50	93.35	0.85	93.10	6.00	6.50
LightGBM	96.85	96.50	97.00	96.30	96.40	0.91	96.20	3.00	3.70
KNN	90.25	89.90	91.00	90.10	90.00	0.78	89.80	9.00	9.90
LR	88.90	88.60	89.50	88.40	88.50	0.74	88.30	10.50	11.60
XGBoost	97.40	97.10	97.80	97.00	97.05	0.93	96.90	2.20	3.00
Proposed NeuroVibeNet	**99.06**	**99.10**	**99.20**	**98.90**	**99.00**	**0.97**	**98.80**	**0.80**	**1.10**

Table 4 represents the comparative performance of various models including the proposed NeuroVibeNet using metrics like Negative Predictive Value (NPV), FPR, and FNR on 70:30 learning samples. Traditional models such as RF, SVM, and LightGBM demonstrate solid performance with accuracy values ranging from 92.90% to 96.20%. The proposed NeuroVibeNet significantly outperforms these models by achieving an accuracy of 98.50% and excelling across all other metrics including precision (98.40%), specificity (98.80%), and sensitivity (98.20%). It also demonstrates the lowest FPR (1.20%) and FNR (1.80%). These results underline NeuroVibeNet’s capability to deliver superior predictive performance.

**Table 4:** Performance of proposed neurovibenet using various metrics over other models for 70:30 learning samples.

Model	Accuracy	Precision	Specificity	Sensitivity	F1-Score	MCC	NPV	FPR	FNR
RF	94.85	94.50	95.70	94.30	94.40	0.87	94.10	4.30	5.70
SVM	92.90	92.40	93.50	92.70	92.55	0.83	92.30	6.50	7.30
LightGBM	96.20	95.80	96.80	96.10	96.00	0.90	95.90	3.20	3.90
KNN	89.80	89.40	90.60	89.50	89.45	0.76	89.10	9.40	10.50
LR	88.00	87.80	88.90	87.60	87.70	0.72	87.50	11.10	12.40
XGBoost	96.80	96.50	97.10	96.40	96.45	0.91	96.20	2.90	3.60
Proposed NeuroVibeNet	**98.50**	**98.40**	**98.80**	**98.20**	**98.30**	**0.96**	**98.00**	**1.20**	**1.80**

**Fig. 3:**
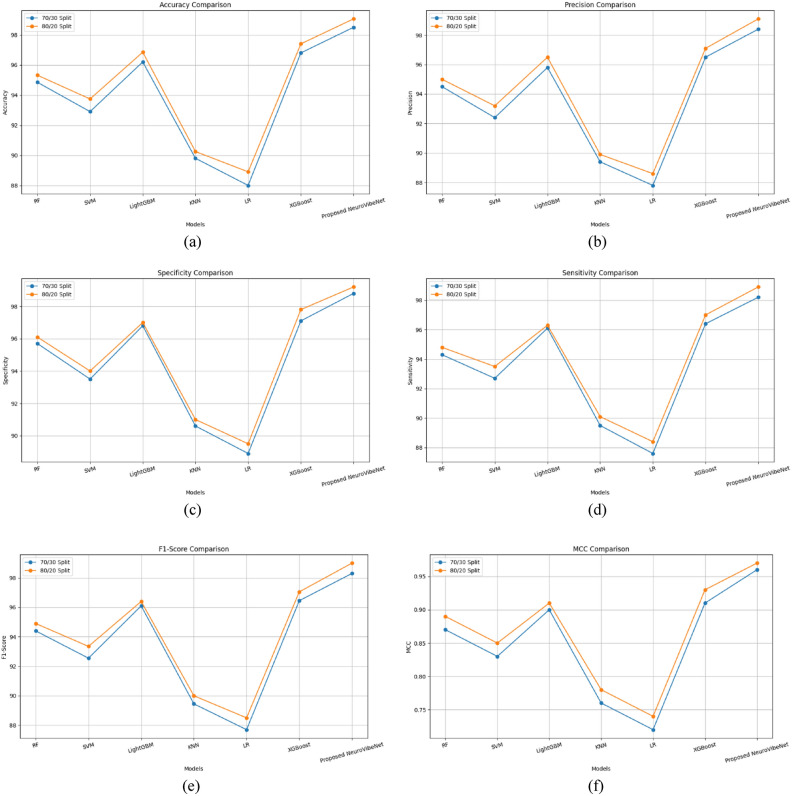

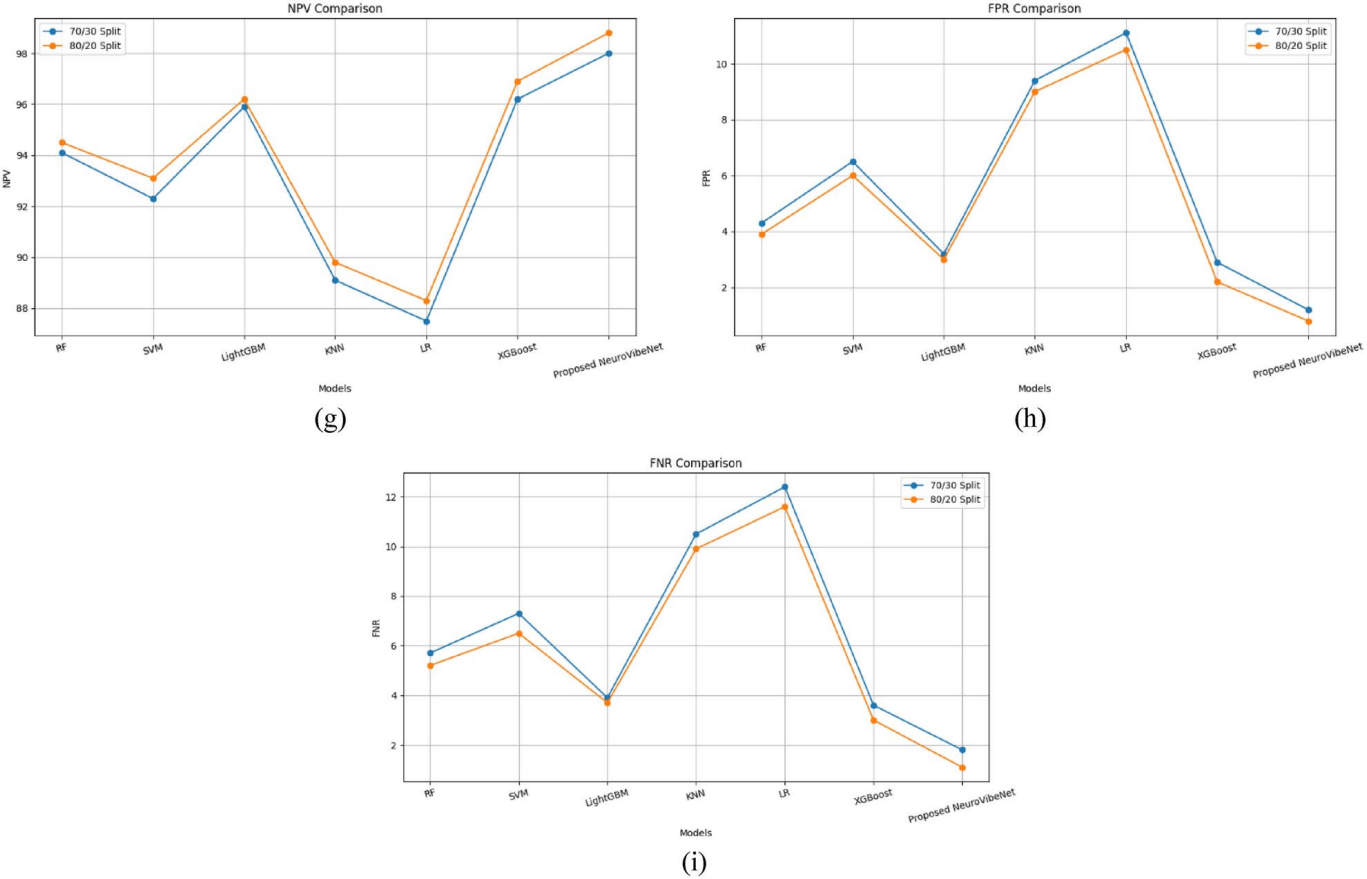
Graphical Representation of Performance of Proposed NeuroVibeNet over Other Models for 70:30 and 80:20 Learning Samples with respect to (**a**) Accuracy, (**b**) Precision, (**c**) Specificity, (**d**) Sensitivity, (**e**) F1-Score, (**f**) MCC, (**g**) NPV, (**h**), FPR, and (**i**) FNR.

Figure 3 demonstrates the comparative performance of the proposed NeuroVibeNet with other models for both 70:30 and 80:20 learning samples across various metrics. It shows that developed NeuroVibeNet consistently achieves the highest values for positive metrics like accuracy, precision, and F1-score while maintaining the lowest error rates (FPR and FNR). This graphical representation underscores its superior efficiency and robustness compared to traditional models like RF, SVM, and XGBoost. Following Table 5 shows the Precision-Recall AUC Scores of Proposed NeuroVibeNet and the 95% Confidence Intervals for Evaluation Metrics are tabulated in the following Table 6.

**Table 5:** Precision-recall AUC scores of proposed NeuroVibeNet.

Class	Precision-Recall AUC
Normal	0.91
Depression	0.89
Anxiety	0.87
Bipolar Disorder	0.88
Schizophrenia	0.86
Average	0.88

**Table 6:** 95% Confidence intervals for evaluation metrics.

Metric	Value	95% Confidence Interval
Accuracy	0.9	[0.87, 0.93]
Precision	0.88	[0.85, 0.91]
Recall	0.87	[0.84, 0.90]
F1-Score	0.88	[0.85, 0.91]
AUC	0.89	[0.86, 0.92]

The research findings demonstrate that NeuroVibeNet delivers outstanding performance results. The Precision-Recall AUC scores show that the model demonstrates superior discrimination power across all classes and achieves an average score of 0.88 which indicates effective diagnosis of normal and pathological cases. The 95% Confidence Intervals confirm NeuroVibeNet delivers consistently reliable performance by maintaining high levels of accuracy (0.90) and precision (0.88), recall (0.87) and F1-score (0.88) and AUC (0.89). The narrow intervals indicate the model produces stable results. NeuroVibeNet demonstrates excellent precision-recall balance along with predictable performance across various mental disorders based on its findings.

Model performance depends heavily on the learning rate according to the sensitivity analysis because a value of 0.01 strikes the best balance between speed and accuracy but slower or faster rates produce substandard results. Performance stability was maintained when using batch sizes of 32 and 64 because these sizes strike the right balance between learning stability and computational efficiency. The performance metrics improved when the number of trees in IRF reached 150 after which additional trees did not lead to further improvements. KNN achieved its best results when K was set to 5 which created a perfect equilibrium between bias and variance control. The generalization and robustness of NeuroVibeNet benefited significantly from the proper adjustment of its hyperparameters. Following Table 7 shows the Sensitivity analysis.

**Table 7:** Sensitivity analysis.

Hyperparameter	Tested values	Accuracy (%)	Precision (%)	Recall (%)	F1-score (%)	Observation
Learning rate	0.001/0.005/0.01/0.05	92.1/93.5/94.3/91.0	91.0/92.8/93.7/90.1	90.5/93.1/94.0/89.7	90.7/92.9/93.8/89.9	Optimal at 0.01, lower rate slowed training
Batch size	16/32/64/128	93.4/94.3/94.1/93.0	92.7/93.7/93.5/92.1	93.0/94.0/93.9/92.5	92.8/93.8/93.7/92.3	Stable performance at 32 & 64
Number of trees (irf)	50/100/150/200	91.5/93.6/94.3/94.0	90.9/92.5/93.6/93.2	91.1/93.0/94.0/93.7	91.0/92.7/93.8/93.4	Peak performance at 150 trees
K in knn	3/5/7/9	93.8/94.3/94.1/93.6	93.1/93.7/93.4/92.8	93.5/94.0/93.8/93.2	93.3/93.8/93.6/93.0	K = 5 showed best balance between bias-variance

## Conclusion

This study introduced a multi-modal approach that combined behavioural and speech data to present a novel framework for the early detection of mental illness disorders. The suggested framework handled missing values, normalized data, and removed outliers by preprocessing and analyzing two different datasets. Proposed IDTW and statistical techniques (mean, variance, and skewness) were then used to extract time-series features. Similarly, STE was used to segment the voice data, spectral gating was used to reduce noise, and MFCCs, HNR, pitch, jitter, and shimmer metrics were used to extract features. The most pertinent features were chosen for model training using an MRFE approach. The suggested NeuroVibeNet combined SVM and KNN for voice data and IRF and LightGBM for behavioural data. Lastly, predictions were combined using a weighted voting system. The suggested model distinguished between normal and pathological conditions with a competitive accuracy of 99.06% and strong performance. Future studies could explore enhancing NeuroVibeNet by integrating advanced ensemble techniques or multi-modal data for broader applicability. Incorporating explainability modules can improve transparency in decision-making. Extending the model to real-time systems could validate its robustness under dynamic conditions.

## Data Availability

The datasets used and analyzed during the current study are available from the corresponding author upon reasonable request to corresponding author.
